# ACE2 decoy receptor generated by high-throughput saturation mutagenesis efficiently neutralizes SARS-CoV-2 and its prevalent variants

**DOI:** 10.1080/22221751.2022.2079426

**Published:** 2022-06-01

**Authors:** Bolun Wang, Junxuan Zhao, Shuo Liu, Jingyuan Feng, Yufeng Luo, Xinyu He, Yanmin Wang, Feixiang Ge, Junyi Wang, Buqing Ye, Weijin Huang, Xiaochen Bo, Youchun Wang, Jianzhong Jeff Xi

**Affiliations:** a Department of Biomedical Engineering, Peking University, Beijing, People's Republic of China; b Division of HIV/AIDS and Sex-transmitted Virus Vaccines, Institute for Biological Product Control, National Institutes for Food and Drug Control (NIFDC), Beijing, People's Republic of China; c College of Chemistry, University of California Berkeley, Berkeley, CA, USA; d Institute of Health Service and Transfusion Medicine, Beijing, People's Republic of China

**Keywords:** SARS-CoV-2, ACE2, high-throughput screening, protein engineering, decoy receptor

## Abstract

The recent global pandemic was a spillover from the SARS-CoV-2 virus. Viral entry involves the receptor binding domain (RBD) of the viral spike protein interacting with the protease domain (PD) of the cellular receptor, ACE2. We hereby present a comprehensive mutational landscape of the effects of ACE2-PD point mutations on RBD-ACE2 binding using a saturation mutagenesis approach based on microarray-based oligo synthesis and a single-cell screening assay. We observed that changes in glycosylation sites and directly interacting sites of ACE2-PD significantly influenced ACE2-RBD binding. We further engineered an ACE2 decoy receptor with critical point mutations, D30I, L79W, T92N, N322V, and K475F, named C4-1. C4-1 shows a 200-fold increase in neutralization for the SARS-CoV-2 D614G pseudotyped virus compared to wild-type soluble ACE2 and a sevenfold increase in binding affinity to wild-type spike compared to the C-terminal Ig-Fc fused wild-type soluble ACE2. Moreover, C4-1 efficiently neutralized prevalent variants, especially the omicron variant (EC ${}_{50}=16$ ng/mL), and rescued monoclonal antibodies, vaccine, and convalescent sera neutralization from viral immune-escaping. We hope to next investigate translating the therapeutic potential of C4-1 for the treatment of SARS-CoV-2.

## Introduction

The ongoing coronavirus disease 2019 (COVID-19) caused by severe acute respiratory syndrome coronavirus 2 (SARS-CoV-2) has emerged as an urgent global health issue [1,2]. As the third outbreak of human coronavirus following SARS and MERS [3, 4], COVID-19 has caused over 500 million infections and 6 million deaths worldwide while emerging SARS-CoV-2 variants have raised global concern [5–7].

SARS-CoV-2 infects human cells with the trimeric spike protein by binding to a receptor named angiotensin converting enzyme II (ACE2) [8–10]. The spike protein consists of two subunits, S1 and S2. Similar to SARS-CoV [11], SARS-CoV-2 virions bind to the extracellular protease domain (PD, residues 19-615) of ACE2 with the receptor-binding domain (RBD) located on the C-terminal domain (CTD) of S1 subunit [12,13]. This interaction was emphasized to be a critical step for viral entry and infection [14]. Although a few studies investigated the spike-ACE2 binding mechanism focusing on the effect of ACE2 mutations on the binding affinity [15–17], they have a couple of inherent limitations: (i) error-prone and randomized synthetic oligonucleotide PCR-based mutagenesis used to establish human ACE2 mutant libraries, thus having biased induction of mutations in A and T, low coverage of mutation type, excessive stop codons or excessive same amino acid mutations with redundant codons; (ii) yeast display system to perform directed evolution, thus taking use of post-translational modifications different from human cells, especially glycosylation which could alter the protein activity including the binding affinity [18–20].

In this work, we employed a saturation mutagenesis approach to comprehensively assess the effects of nonsynonymous mutations on the binding affinity of SARS-CoV-2 spike RBD (S-RBD) with human ACE2-PD. A mutagenesis library of $10^{7}$ positive cells was obtained as a cell library through FACS, and the mutational landscape influencing the interaction between S-RBD and ACE2-PD was generated. Furthermore, the soluble form of ACE2 (sACE2) has been engineered to bind to S-RBD, thus preventing viral infection [21,22]. Based on our mutation analysis, we further engineered a new ACE2 decoy receptor with a high S-RBD affinity ( $K_{D}$ = 0.44 nM). This new engineered ACE2 decoy receptor demonstrated remarkable neutralization capacities against diverse prevalent SARS-CoV-2 mutants, especially in combination with monoclonal antibodies that may lose protection from resistant strains.

## Results

### A saturation mutagenesis approach to comprehensively study the affinity of ACE2 PD to SARS-CoV-2 RBD

**Figure 1.**
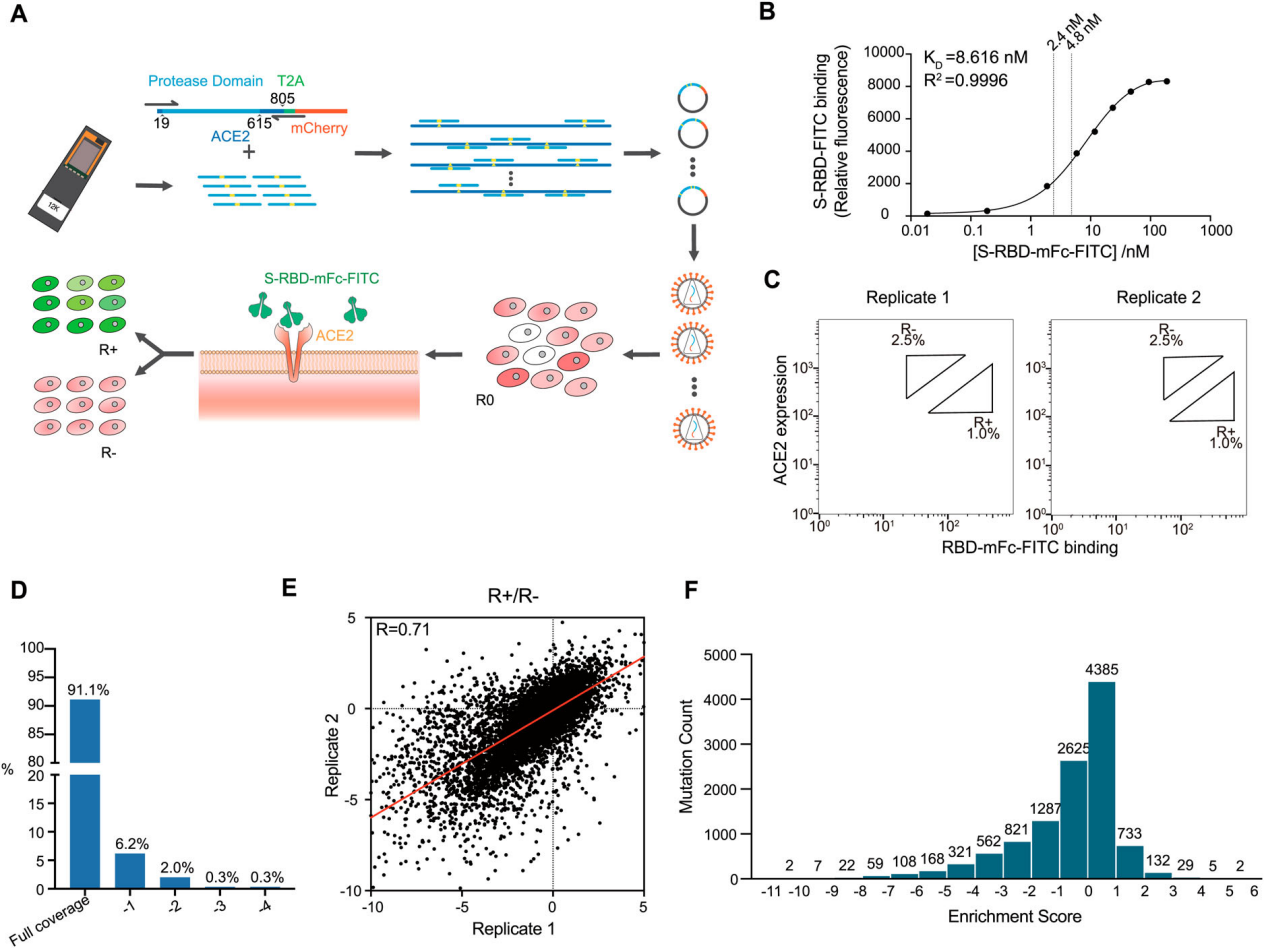
Generation of ACE2-PD saturation mutagenesis library and high-throughput screening. (a) Approach for establishment and screening of mutagenesis library. Oligo-synthesized saturation mutagenesis primers were used to construct a full-length human ACE2 lentiviral vector. Lentivirus ( MOI<0.3) was transducted in HEK-293T cells and a mammalian cell mutant library (R0) was obtained after one round of FACS. After the incubation with SARS-CoV-2 S-RBD-mFc-FITC, a second round of FACS yielded RBD positive and negative groups (R+, R−). (b) FITC signal for different titres of WT-ACE2-expressing HEK-293T cells was added to RBD-mFc-FITC in growth medium. Bound RBD-mFC-FITC was measured by flow cytometry. Dashed lines indicate the different concentrations of two replicates. (c) FACS imaging for the cell mutant libraries in different concentrations of RBD protein: 2.4 nM RBD-mFc-FITC (left) and 4.8 nM RBD-mFc-FITC (right). (d) The ratio of amino acids' R+ and R− sequencing coverage. Full coverage indicated all intended mutant sites of amino acids were covered. −1 means 1 mutant site was missing, −2 means 2 were missing, and so forth. (e) Enrichment scores for ACE2 mutations highly agreed between two independent FACS experiments (Pearson's *r* = 0.71). (f) The distribution of enrichment scores of all detected mutations. The total number of mutant sites was 11,268 from 2 replicates (the mutant site may be missing in 1 replicate).

We developed a human single cell-based assay to investigate the binding affinity of ACE2 PD to SARS-CoV-2 RBD with the use of surface display of mutagenized PD library combined with fluorescence-activated cell sorting (FACS). First, we designed and synthesized a comprehensive library of full-length PD domain (residue 19-615), in which each amino acid in the PD domain mutated into 19 other natural amino acids by their most frequently used codons. In brief, we synthesized 11,343 mutation primers on an integrated microarray (Figure 1a and Supplementary Figure 1). Through tuning the concentration of those mutation primers in a PCR, a library of mutated ACE2 containing an average of 1.8 custom-designed point mutations was efficiently generated (see Supplementary Figure 2a). ACE2 N-terminal 18-aa signal peptide was retained for the transportation of ACE2 to plasma membrane [23]. The library was inserted into the 5' end of the mCherry encoding region of the restriction endonuclease vector and connected with T2A tag (Figure 1a). The constructed plasmids were transformed into *E. coli* cells and a mutant library of over $10^{7}$ mutants was obtained.

To minimize the background noise, we collected a group of cell lines and examined the expression level of endogenous ACE2 in comparison with one of the lentivirus-transfected ACE2. As evidenced by real-time PCR, HEK-293T cell line had a relatively low endogenous expression level (1/2309, Supplementary Figure 2b). Mutagenesis plasmid library was packaged into lentivirus and then expressed in HEK-293T cells in less than 0.3 multiplicity of infection (MOI) to yield no more than one copy of ACE2 per cell. Infected cells were incubated with a probe, which consisted of SARS-CoV-2 spike protein RBD conjugated with fluorescein isothiocyanate (FITC) (Figure 1a). To improve the detection sensitivity of the probe, SARS-CoV-2 spike protein was also fused to a mouse C-terminal IgG Fc (mFc) to form dimers due to the mFc interaction [24]. Both the expression level of ACE2 and the RBD-FITC affinity were confirmed by fluorescent microscopy (see Supplementary Figure 3).

To achieve the best signal-to-noise ratio, a dose-binding affinity curve was drawn and two concentrations in a sensitive range were chosen for independent replicates (Figure 1b). As shown in flow cytometry, two subsets of the ACE2-positive population were collected: the top 1% (RBD-high sort, defined as R+ group) and the top 2.5% (RBD-low sort, defined as R− group) based on the fluorescence of bound RBD-mFC-FITC relative to ACE2 surface expression, respectively (Figure 1c). Total RNA was extracted from the pre-screening cell library (defined as R0 group), R+ and R-groups, followed by deep sequencing. It was found that more than 99.9% (11,338/ 11,343) of designed mutations were present in the R0 group. The enrichment scores of nonsynonymous mutations were set as the log ${}_{2}$-scaled ratio of the frequencies of transcripts between the sorted populations (R+/R−). Mutation reads from both R+ and R− groups in two biological replicates cover more than 99.3% of all designed mutations (11,268/ 11,343, Figure 1d, Supplementary Figure 4).

We evaluated reproducibility using two independent FACS experiments as biological replicates. Enrichment scores were generated for 11,268 nonsynonymous mutations and were shown to be highly correlated between independent experiments (Pearson's *r* = 0.71, Figure 1e). The distribution of enrichment scores centred around 0 (Figure 1f). The majority of enrichment scores (62%, 7010/ 11,268) lay in the range of −1 to 1, indicating that mutation had a relatively low influence on the binding affinity. About 30% (3357/ 11,268) of enrichment scores were less than −1, indicating that these mutations had a significant deleterious impact on the binding, whereas 8% (901/ 11,268) of enrichment scores were greater than 1, showing that these mutations may increase the binding affinity.

### Mutation-affinity insights by ACE2-PD saturation mutagenesis

**Figure 2.**
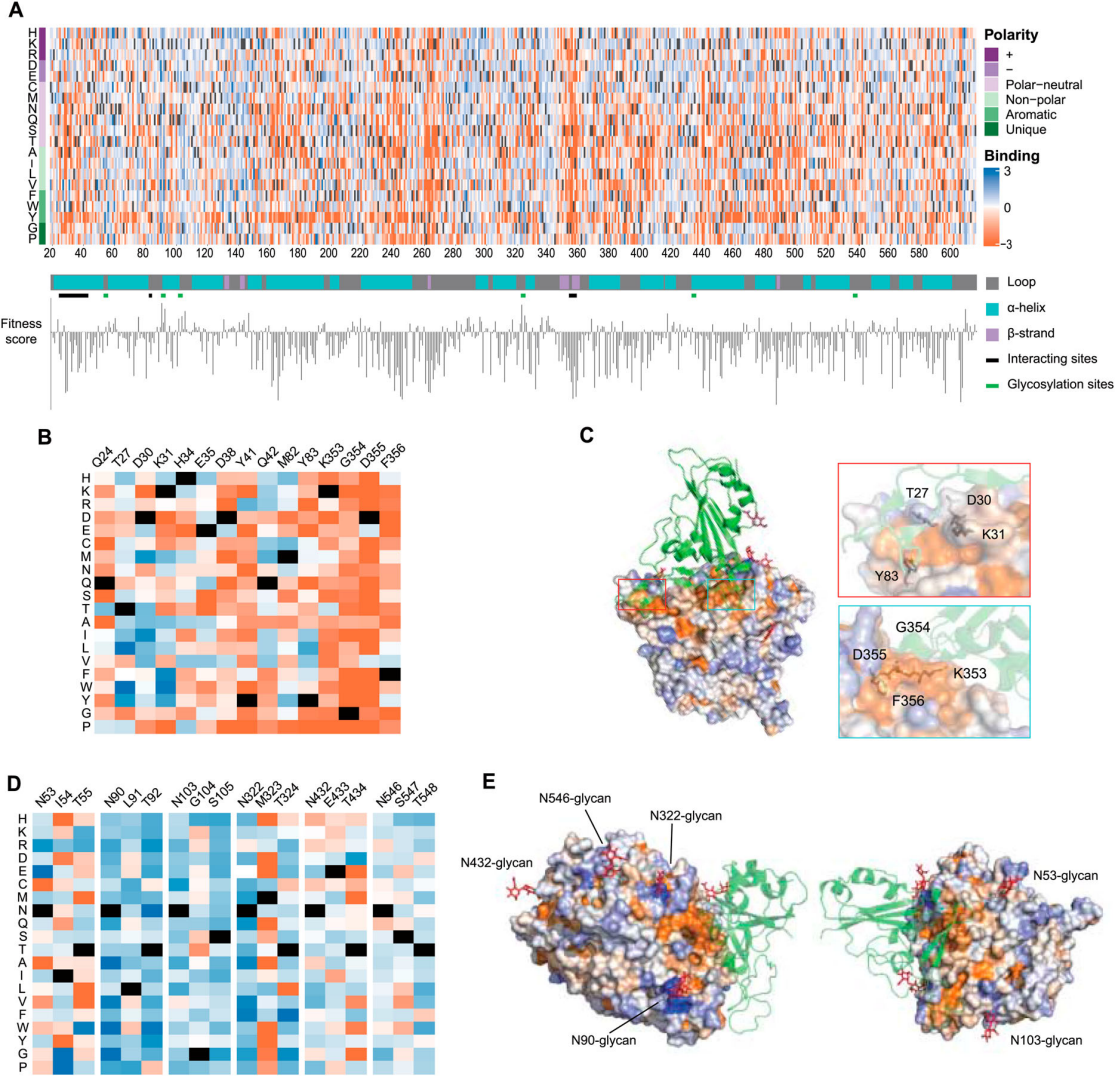
Full-length ACE2-PD enrichment heatmap. (a) ACE2-PD saturated mutagenesis heatmap and classification. Fitness scores based on the average Log2 enrichment ratios from two replications of the RBD sorts were plotted from depletion or deleterious (orange) to enriched (blue). Positions on ACE2 protein were shown on the horizontal–vertical axis, and amino acid substitutions are indicated on the vertical axis. Magnified views of ACE2 positions that directly interact with RBD (b) and glycosylation sites (d). Colouring schemes were the same as in (a). (c) Fitness scores were mapped to the structure (PDB: 6M0J) of RBD (green cartoon)-bound protease domain (surface). Residues conserved for RBD binding were shown in orange; mutationally tolerant residues were in pale colours; residues that were hot spots for enriched mutations were in blue. (e) Views looking down onto the ACE2 glycosylation sites (PDB: 6M17). The colouring scheme was the same as in (a).

We aligned the enrichment scores of all collected 11,268 mutations by both position and amino acid properties to generate a full-length ACE2 PD enrichment heatmap (Figure 2a, interactive version: https://github.com/JillFeng123/SARS-CoV2-rACE2-RBD_Affinity_Map). We first analysed the enrichment score of every amino acid on the PD domain and the secondary structure types of the domain, which were classified into alpha-helix, beta-strand, and loop by applying the STRIDE algorithm (Figure 2a). To quantitatively analyse the effect of different amino acids on binding affinity, we defined the fitness score of a position as the average enrichment score of mutations to all 19 amino acids at a given position. We found that the average fitness score for positions in alpha-helix, beta-strand, and loop are −0.73, −1.24, and −0.48, respectively. The loop had significantly higher ratio of positive to negative enrichment scores than alpha-helices (p=0.0024) and beta-strands (p=0.019) (Figure 2a and Supplementary Figure 5). Thus we concluded that amino acids on alpha helices and beta strands tended to be more sensitive to mutations than the ones on loops.

We next characterized two regions that significantly affect the binding affinity (the direct 43 interaction region and glycosylation sites). First, the direct interaction region for S-RBD binding on ACE2 carried mostly deleterious mutations, which was consistent with the findings from the crystal structure of ACE2-RBD complexes (enrichment score <1) (Figure 2b and c and Supplementary Figure 6a). In particular, nearly all mutation types through ACE2's 353–356th amino acids were significantly deleterious, whereas the positions on alpha1 (Q24, T27, D30, K31, H34, E35, D38, Y41 and Q42) of ACE2 PD had various degrees of mutational tolerance. We then examined the effect of ACE2 glycosylation on ACE2-RBD binding affinity. ACE2 is a highly glycosylated protein with 6 N-glycosylation sites, namely N53, N90, N103, N322, N432, and N546 [25,26]. In N-glycosylation, the protein's asparagine residues are connected with oligosaccharide molecules when the two amino acids downstream of asparagine follow a certain sequon form (N-X-T/S, while X ≠ P). N53-glycan is close to the ACE2-RBD binding interface and plays an important role in stabilizing the dimer interface of ACE2 [27]. However, the mutants removing N53-glycan were not enriched in RBD binding screening assays (Figure 2 d,e). We assumed that the mutants resulted in two opposite effects: the mutants are likely to increase the accessibility of ACE2 surface to S-RBD, whereas they impair the stability of ACE2 dimer. Mutations that removed glycosylation in N432 and N546 were not favoured (Figure S6b), possibly because they were distant from protein interaction sites. N90, N103, and N322-glycans hover over a fraction of the binding surface [28,29]. Mutations that remove glycosylation at these glycan sites were significantly enriched (Figure 2d,e): the fitness scores of both asparagine (N) and threonine (T)/serine(S) of the three glycan sites were positive (see Supplementary Figure 6b), indicating that de-glycosylation boosts binding by reducing steric strain.

We further identified 118 mutations in our library potentially introduce N-glycosylation sites (N-X-T/S sequon forms, X ≠ P). Among the 118 mutations, 49.2% have enrichment scores less than −1 (58/118); 49.2% have enrichment scores between −1 and 1 (58/118), showing the insignificant influence on binding affinity and protein stability. Only 1.6% of mutations have enrichment scores greater than 1 (2/118), but both mutations are at positions afar from RBD-ACE2 interaction sites (see Supplementary Figure 7). Mutations at these two positions had different enrichment effects when mutated to T and S, indicating that enrichment at these positions was unlikely caused by glycosylation. Of these 118 mutations, 22 appeared at interacting sites. 14 of 22 had enrichment scores ranging from −1 to 1, and 8 were less than −1, implying that introduced N-glycans on ACE2 were potentially detrimental to the binding of ACE2 with RBD.

We chose single amino acid substitution mutations that scored highest from the ratio of frequency of transcripts between the sorted populations (R+/R−) of two independent biological replicates. We transiently transfected these variants and mCherry in HEK-293T cell line. Likewise, after incubating the RBD-mFc-FITC probe, we used flow cytometry to quantify FITC brightness. We selected single amino acid variant candidates for combination, namely, D30I, L79W, T92N, N322V, and K475F (see Supplementary Figure 8).

A group of HEK-293T cell lines expressing single amino acid substitutional ACE2s was collected to characterize their binding affinity for S-RBD (Figure S9). The D30 amino acid residue forms electrostatic interactions with RBD through a salt bridge with RBD-K417 [25,30]. D30I scored the highest on enrichment among D30 substitutions. The hydrophobic packet comprising L79W, Q24, M82, and Y83 formed hydrophobic interactions with RBD F486 [12] (see Supplementary Figure 9). The L79W variant showed the highest enrichment score among L79 substitutions. K475F might reduce repulsion from M474, since both F and M are hydrophobic residues. Therefore, hydrophobic stacking due to K475F mutation might stabilize the helix. T92N and N322V removed the glycans at N90 and N322, respectively, therefore might reduce steric hindrance to the binding.

We used the soluble segment of ACE2 (sACE2, residue 1-740) to engineer and purify mutated ACE2 proteins. A mouse IgG-Fc peptide was fused to the C-terminal of sACE2s to generate ACE2 mutant decoy receptors (sACE2-Fc). To characterize the binding affinities of ACE2 mutant decoy receptors for spike RBD, we used surface plasmon resonance where His-tagged RBD was immobilized as a ligand, and the association and dissociation kinetics of sACE2-Fc were determined. We evaluated 7 mutant sACE2-Fc proteins including one containing 5-site mutations (C5), two containing 4-site mutations (C4-1 and C4-2), three containing 3-site mutations (C3-1, C3-2, and C3-3, respectively) and a WT-sACE2-Fc. All combinatorial mutants were shown in Supplementary Table 1. As shown in Supplementary Figure 10(a–d), the $K_{D}$ value of C5 was determined to be 0.60 nM. The $K_{D}$ value of mutants C3-1, C3-2, and C3-3 were 0.93 nM, 0.53 nM, and 0.58 nM, respectively. C4-1 and C4-2 showed the lowest $K_{D}$ value among the multi-mutants, both around 0.45 nM (see Supplementary Figure 10e,f). The two variants had binding affinity comparable to monoclonal antibodies for RBD. Compared to WT-sACE2-Fc ( $K_{D}=3.13$ nM, Supplementary Figure 10g), our engineered sACE2-Fc improved the affinity by about seven times to SARS-CoV-2 spike RBD.

### Modified ACE2 decoy receptor efficiently neutralizes SARS-CoV-2 pseudotyped viruses

**Figure 3.**
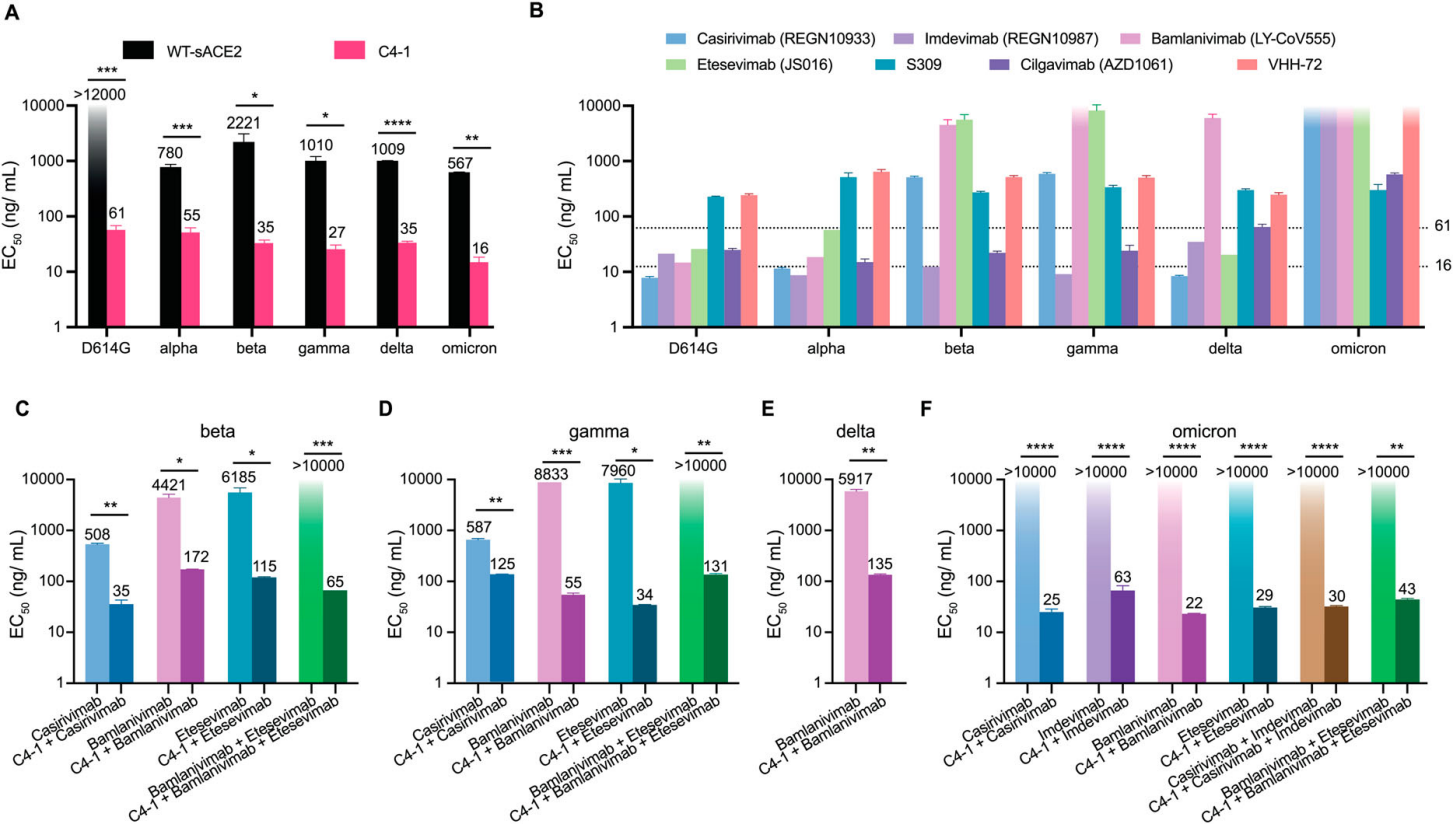
Analysis of antigenicity of SARS-CoV-2 variants using a panel of engineered ACE2 and neutralizing monoclonal antibodies. (a) The inhibition effect of WT-sACE2 (black) and C4-1 (pink) in SARS-CoV-2 variants entrance into ACE2 expressing HEK-293T cell lines. (b) The inhibition effect of monoclonal antibodies. Dashed lines indicate the neutralizing range of C4-1. Therapeutic effects for cocktails of C4-1 and monoclonal antibodies against beta (c), gamma (d), delta (e), and omicron (f) variants. Unpaired *t*-test was used to analyse differences between groups. *p<0.05, ** p<0.01, ***p<0.001, ****p<0.0001. EC ${}_{50}$ was calculated based on all dilutions of soluble ACE2 protein. The limit of EC ${}_{50}$ detection was 0–12000 ng/mL for panel A and 0–10000 ng/mL for panel b, c, d, e, and f. All results were obtained from three independent experiments. In each experiment, the infection assay was performed in duplicate wells. Data are mean ±SEM, *n* = 2 replicates.

We picked C4-1 as an affinity-enhanced sACE2-Fc mutant and evaluated its efficacy in neutralizing SARS-CoV-2 infection using a pseudotyped-VSV reporter assay, whose envelope plasmid encodes the spike protein [31,32]. We examined the neutralizing effect of C4-1 to 6 spike variants, namely, D614G, alpha (B.1.1.7), beta (B.1.351), gamma (P.1), delta (B.1.617.2), and omicron (B.1.1.529.1). The half maximal effective concentration (EC ${}_{50}$) of C4-1 towards the D614G spike decreased more than 200 times compared to WT sACE2, indicating significantly higher neutralizing efficacy (Figure 3a). C4-1 achieved greater neutralization than the WT sACE2 protein on prevalent coronavirus variants as well, increasing neutralizing efficiency by 14 -- 63 times, respectively (Figure 3a and Supplementary Figure 11). WT sACE2 showed the lowest EC ${}_{50}$ neutralizing omicron variant, indicating that the omicron spike has strong cell receptor binding affinity. C4-1 also efficiently neutralized omicron pseudovirus with the lowest EC ${}_{50}$ at 16 ng/mL.

Seven reported neutralizing monoclonal antibodies: casirivimab, imdevimab, bamlanivimab, etesevimab, S309, cilgavimab and VHH-72, were examined using the same neutralization assays [33–38]. We found that all seven neutralizing antibodies showed decreased or absent neutralizing protection against prevalent variants (Figure 3b and Supplementary Figure 12). Notably, casirivimab/imdevimab (REGEN-COV) and bamlanivimab/etesevimab are FDA EUA-authorized monoclonal antibody cocktails for COVID-19 [33,34]. Also, casirivimab, bamlanivimab, and etesevimab lost their sensitivity to the beta and gamma pseudotyped viruses, and bamlanivimab neutralization against delta variant was significantly impeded as well. Furthermore, casirivimab, imdevimab, bamlanivimab, and etesevimab failed in neutralizing the omicron variant (Figure 3b and Supplementary Figure 1a). Compared to tested antibodies, C4-1 showed greater neutralization efficiency and stable neutralization for prevalent variants.

Furthermore, C4-1 and monoclonal antibodies were mixed to neutralize escape variants. C4-1/casirivimab cocktail (1:1) obtained suitable neutralization against the pseudotyped virus of both the beta and gamma variants while maintaining high neutralizing activity for other variants (Figure 3c,d and Supplementary Figure 13a). C4-1 also compensated the immune escape of bamlanivimab, etesevimab, or bamlanivimab/etesevimab against beta and gamma. When proportionally mixed with C4-1, bamlanivimab, etesevimab, and bamlanivimab/etesevimab re-established effective neutralization (Figure 3 c,d and Supplementary Figure 13b). Besides, C4-1/bamlanivimab demonstrated effective neutralization against delta (Figure 3e). For the most widely prevalent variant, omicron, monoclonal antibodies/cocktails showed a significant neutralizing efficiency increase when (1:1) mixed with C4-1 (Figure 3f and Supplementary Figure 13a,b). Therefore, C4-1 not only produced a strong neutralizing effect on various prevalent variants but also restored neutralizing effect when mixed with monoclonal antibodies that lost neutralizing effect against resistant variants.

**Figure 4.**
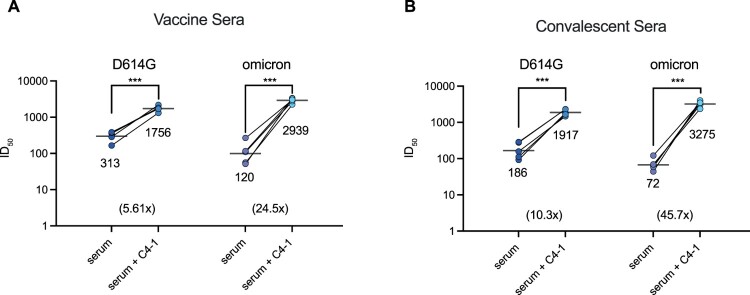
Enhanced serum neutralization against D614G and omicron pseudotyped virus by C4-1. The indicated pseudotyped viruses were incubated with dilutions of serum derived from individuals vaccinated with Rong'an vaccine or vaccine C4-1/serum mixture (a) and serum from COVID-19 patients or patients convalescent C4-1/serum mixture (b). The lines indicate the geometric mean ID ${}_{50}$. The limit of ID ${}_{50}$ detection was >30. Paired *t*-test was used to analyse differences between groups. ***p<0.001. Data are mean ±SEM, *n* = 2 replicates.

Sera isolated from SARS-CoV-2 patients and vaccinated individuals are believed to contribute to protection from infection and reinfection. However, studies have recently shown that serum samples collected from vaccine recipients failed to neutralize emerging SARS-CoV-2 variants [39–41]. To determine whether C4-1 could restore the neutralizing ability of vaccine serum and convalescent patient serum, C4-1 and serum samples were proportionally mixed (100 μg/mL: undiluted serum = 1: 1) to test the combinational neutralization potency against SARS-CoV-2 D614G and omicron pseudotyped-VSV virus. For both D614G and omicron pseudotyped viruses, the combination of C4-1 and vaccine serum produced a significant increase in neutralization potency (p<0.001). The neutralizing potency of the C4-1/vaccine serum mixture (mean 50% inhibitory dose, ID ${}_{50}=1756$) was 5.61 times higher than that of serum alone (mean ID ${}_{50}=313$). The same trend also appeared in the omicron assay. The neutralizing potency of the C4-1/vaccine serum mixture was 24.5 times higher than that of serum alone, ID ${}_{50}$ increased from 120 to 2939, growing 24.5 times (Figure 4a and Supplementary Figure 14a,b). Convalescent sera were obtained from SARS-CoV-2 infected patients from March to October, 2020. For both D614G and omicron pseudotyped viruses, the neutralization potency of convalescent sera mixed with C4-1 was also significantly improved (p<0.001). The neutralizing potency of the C4-1/convalescent serum mixture (mean ID ${}_{50}=1917$) was 10.3 times higher than that of serum alone (mean ID ${}_{50}=186$). In the omicron assay, serum ID ${}_{50}$ increased from 72 to 3275, growing 45.7 times (Figure 4b and Supplementary Figure 14a,b). The ID ${}_{50}$ value of the C4-1/serum mixture was higher against omicron than D614G (Figure 4a,b), confirming that C4-1 had the highest neutralization potency for omicron among the tested circulating strains (Figure 3a). These results indicate the C4-1's efficacy and potential in neutralizing emerging SARS-CoV-2 variants.

## Discussion

Protein engineering by mutagenesis through directed-evolution has played an important role in the life sciences [42,43]. Degenerate codes (NNN, NNK, NNS) have been widely used to obtain saturated protein mutagenesis [44–47], but the use of degenerate codons necessitates codon redundancy, uneven distribution of amino acid mutations and nonsense mutations. Recent advances in microarray-based DNA synthesis changed this paradigm [48,49]. In the present study, we harnessed the DNA microarray technology to circumvent the disadvantages of oligo synthesis. Without the limitations mentioned above, we generated a saturation mutagenesis library of ACE2-PD using an oligonucleotide pool featuring precisely designed amino acid mutations. Saturated mutation for every amino acid in a 597-amino acid sequence was obtained, with a 99.9% coverage of all intended variants. Additionally, introducing mutations at multiple positions through PCR mutagenesis further increases the diversity of the mutagenesis library. Thus our method integrated rational design and saturation mutagenesis to achieve greater realistic variant coverage and efficiency.

We employed such a saturation mutagenesis approach to comprehensively assess the effects of nonsynonymous mutations on the binding affinity of S-RBD with human ACE2-PD. The single-cell screening assay employed here also showed a few advantages over recently reported works. First, a C-terminal mCherry was fused to the ACE2 gene through a T2A motif to indicate the expression level of the ACE2 mutants, rather than the use of fluorescent secondary antibodies to quantify the ACE2 expression [15,17], which generated high noise caused by indels and incomplete ACE2 variants. Second, human cell-based screening is more suited for understanding the impact of ACE2 variants on viral affinity and biologics development, since yeast surface display has inherent limitations in post-translational modification [16,50]. In addition, our strategy can be easily adapted for studying other viral infections via protein–protein interaction.

We identified key positions that affect the binding affinity of ACE2 through a single-cell analysis of protein binding affinity, thereby obtaining a complete ACE2 PD amino acid mutation map. Positions on alpha helices and beta strands tend to be more conserved, while positions on loops seem to be more tolerant to mutations. The conservativeness of different amino acid residues that directly interact with RBD was demonstrated. We also found that glycans play a very important role in ACE2-RBD binding. The ACE2-PD saturation mutagenesis heatmap showcases the negative impact of N90 and N322 glycans for spike binding. Removing these two glycans increased ACE2-spike binding affinity. Most N90 mutations were enriching possibly because the N90-glycan shielded ACE2 from RBD binding [28]. Although N103 and N322 glycosylation sites are not on the ACE2-RBD binding interface, glycans formed at these sites orient toward RBD [28,29]. This clash may produce steric hindrance during ACE2-RBD binding. The identification of new glycosylation positions was likely due to the use of human cell culture and full coverage of the protein, whereas previous studies used yeast with distinct glycosylation patterns or incomplete coverage. We summarized recently reported amino acid alterations on ACE2 that improve RBD binding efficiency in Supplementary Table 2. Most of these previously reported ACE2 mutations were on N-terminal alpha helixes (I19-Q81). We identified two key sites, D30I and L79W. Combined mutations at these two positions and glycosylation sites allowed us to generate high-affinity ACE2 mutants.

Researchers and industrial collaborators worldwide have designed and manufactured vaccines [51,52] and monoclonal antibodies [33,34] for specific variants of the SARS-CoV-2 virus. However, since coronavirus replication is RNA-dependent and thus error-prone [53], new variants evolve rapidly with higher infection rate and/or mortality rate. Thus novel therapeutic modalities are urgently needed for dealing with emerging variants and their immune evasion from previously administered therapeutics. C4-1 is a potential candidate with two powerful advantages. First, C4-1 exhibits high resistance to viral escape mutation, since mutations that reduce affinity of the soluble decoy will likely also decrease affinity for the wild-type receptor on host cells. Second, C4-1 elicits low immunogenicity due to ACE2 endogenous expression. Although wild-type ACE2 is currently in Phase 2 clinical trial [54], its affinity for SARS-CoV-2 spike is limited [10,16,55]. Meanwhile, C4-1 showed improved spike affinity compared to sACE2 and broad neutralization against SARS-CoV-2 variants. The combination of C4-1 with nAbs could keep the high neutralization capacity of both components against certain SARS-CoV-2 variants, while maintaining the C4-1 neutralization capacity against other variants if the antibody loses neutralization. This indicates that C4-1 could have high therapeutic potential both administered independently or combined with antibodies. Due to its high binding ability and spectral effectiveness to SARS-CoV-2 variants, C4-1 also has the potential to be used for SARS-CoV-2 antigen detection. Thus we suggest that C4-1 has high potential in the design of new treatment modalities, disease prevention, and control.

In summary, we constructed a saturated mutagenetic library of ACE2-PD to analyse the effects of ACE2-PD mutation patterns on its binding to RBD. We identified key amino acid residues, glycosylation sites, and secondary structure types involved in ACE2-RBD binding. Multiple engineered ACE2 mutants were characterized based on our mutation enrichment heatmap were characterized. The most potent variant improved spike binding affinity 7-fold compared to WT-sACE2-Fc. We developed an sACE2 decoy, C4-1, based on this variant with neutralization that rivals clinically used monoclonal antibodies of SARS-CoV-2.

For both the wild-type virus and prevalent strains, we achieved greater viral neutralization through administering sACE2 protein alone or in combination with monoclonal antibodies, vaccine, and convalescent sera. Therefore, C4-1 shows great therapeutic potential to be translated into a neutralizing drug to inhibit viral infections for rapidly evolving variants.

## Materials and methods

### Plasmids

The human ACE2 (NM_001371415) coding region was inserted into a lentivirus transfer vector using the NotI and EcoRI sites under the control of the CMV promoter with T2A and mCherry by Gibson Assembly to obtain the plasmid pLV03 hACE2. For the generation of the expression plasmids for the SARS-CoV-2 spike, the coding sequence of synthetic, codon-optimized SARS-CoV-2 spikes were cloned into the expression vector. Compared to NCBI Reference Sequence: YP_009724390.1, for different variants of SARS-CoV-2 spikes, mutations were introduced as: D614G (D614G), alpha (69-70del, 144del, N501Y, A570D, D614G, P681H, T716I, S982A, D1118H), beta (L18F, D80A, D215G, 242-244del, K417N, E484K, N501Y, D614G, A701V), gamma (L18F, T20N, P26S, D138Y, R190S, K417T, E484K, N501Y, D614G, H655Y, T1027I, V1176F), delta (T19R, G142D, 156-157del, R158G, L452R, T478K, D614G, P681R, D950N), and omicron (A67V, 69-70del, T95I, G142D, V143del, Y144del, Y145del, N211del, L212I, ins214EPE, G339D, S371L, S373P, S375F, K417N, N440K, G446S, S477N, T478K, E484A, Q493R, G496S, Q498R, N501Y, Y505H, T547K, D614G, H655Y, N679K, P681H, N764K, D796Y, N856K, Q954H, N969K, L981F)

### Cell culture

HEK-293T, A-375, and Huh-7 cells were cultured in Dulbecco Modified Eagle Medium (DMEM, Thermo Fisher) supplemented with 10% fetal bovine serum and 100 U/mL penicillin/streptomycin at 5% CO ${}_{2}$, 37 ${}^{\circ }$ C. Cell culture media and supplements were obtained from Life Technologies, Inc.

### Proteins, monoclonal antibodies, and sera

Purified recombinant proteins C3-1, C3-2, C3-3, C4-1, C4-2, C5, WT-ACE2-Fc, and S-RBD-mFc-FITC were ordered according to amino acid sequences from Suzhou SGE Biotech. WT-sACE2 was purchased from Sino Biological, Inc. A total of seven neutralizing monoclonal antibodies against SARS-CoV-2 S protein were purchased from Wuhan Chemstan Biotechnology Co., Ltd. Vaccine sera from individuals vaccinated with an SARS-CoV-2 inactivated vaccine (Rong'an Biological, Novel Coronavirus inactivated vaccine (Vero cells), clinical

trial registration number: ChiCTR2100050024) were obtained 28 days after the first dose. Convalescent serum samples from convalescent COVID-19 patients who were infected with the Wuhan-Hu-1 variant were collected from March to October 2020 by the Institute of Translational Medicine, First People's Hospital of Chenzhou 3 months after recovery.

### Generation of the ACE2-PD amino acid mutagenesis library

Using the human ACE2 DNA sequence as the template, each amino acid of the PD domain (S19-D615) was designed to mutate into other 19 natural amino acids to construct mutagenesis primers. The upstream and downstream of the pre-mutation site are 21 nt long, respectively. The amino acid triplet codons at the pre-mutation site were replaced with the resultant amino acid with the most frequently used codons in human. Each end of the primer is a recognition site for the BspQI restriction enzyme (NEB) and a fixed-length adaptor for amplification. Oligonucleotides were synthesized with a 12k microreactor chip using a high-throughput oligonucleotide synthesizer (CustomArray B3). The synthesized oligonucleotides are listed in Supplementary Table 3.

To obtain the mutagenesis primer pool, the oligonucleotide mix library taken from the microreactor chip was amplified using KAPA HiFi HotStart ReadyMix (Roche), with the upstream and downstream adaptors as primers. The resulting PCR product was then digested with endonuclease BspQI to obtain the double-stranded mutagenesis primer library.

Mutagenesis primers were integrated into ACE2 plasmids through a 20-cycle TouchDown PCR. The PCR product was ligated into the pLV03 plasmid using Gibson Assembly (NEB) to obtain the circular mutagenesis library, and then packaged into lentivirus and transfected into HEK293T cells. The method for mutagenesis library construction was demonstrated in Supplementary Figure 1.

### Flow cytometry

HEK-293T cells were analysed by flow cytometry 48 h after lentivirus transfection. For RBD binding to ACE2, ACE2-expressing cells were washed with ice-cold PBS-BSA, and incubated for 30 minutes on ice with a medium containing recombinant spike RBD fused to a C-terminal mouse IgG-Fc (mFc) and Fluorescein Isothiocyanate (FITC) modification. Cells were washed with PBS and analysed on a Beckman MoFlo. Data were processed with FlowJo.

### Data curation and analysis

Total RNA was isolated from R0, R+, and R− with TRNzol reagent (TIANGEN), and cDNA was reverse transcribed using FastQuant RT Kit (TIANGEN). The coding sequences of ACE2 PD were amplified as 10 fragments. Flanking sequences on the primers added adapters to the ends of the products for annealing to Illumina sequencing primers, unique barcoding, and for binding the flow cell. Amplicons were sequenced on an Illumina NovaSeq 6000 using a 2×250 nt paired-end protocol. The raw variant read count files were collected and analysed with a python-based method (see data availability). log ${}_{2}$-median transformed to calculate enrichment scores. Standard errors were calculated using GraphPad to calculate confidence intervals. There were two selection criteria for high-confidence variants. If the 95% confidence interval of the enrichment score was less than or equal to 1.42, the variant was considered high confidence. If the 95% confidence interval was less than 1.42 but the scores from each biological replicate were concordant, the variant was also considered high confidence. For all subsequent data analysis, we used median as opposed to mean as a summary statistic because the mutational enrichment scores follow a modal rather than normal distribution.

### Protein features and clustering model

Relative solvent exposure was obtained from GETAREA. Secondary structural information of ACE2 positions was obtained using STRIDE. Clustering was performed with the ComplexHeatmap package in R, method = “ward.D”, distance = “Euclidian”. Positional fitness scores were calculated as the average enrichment score for a position. Structural images were generated based on known crystal structures from PDB: 6M0J [12]. The size of amino acids was obtained from IMGT.

### Surface plasmon resonance test for ACE2 binding

The protein–protein interaction between ACE2 and spike-RBD was quantitatively measured by their binding ability on a CM5 chip, using Biacore 2000 instrument. 1× HBS-EP (Cytiva) was used as the running buffer. RBD-Fc (20 ng/μL) was dissolved in sodium acetate buffer (pH 4.5), and then immobilized as the ligand on the chip, with an 60 RU increase in resonance signal. Several kinds of mutant sACE2-Fc were employed as the analyte, with twofold gradient dilutions of concentration ranging from 100 to 1.56 nM. The flow rate was set at 45 μL/min, with 180 s for binding and 1800 s for dissociation. At the end of a running circle, the sensor chip was regenerated with imidazole for 30 s.

### Pseudotyped VSV neutralization assay

Pseudotyped viruses of SARS-CoV-2 and its variants were constructed according to previous studies [31,32]. The HEK-293T cells were adjusted to concentrations of $5\times 10^{5}$– $7\times 10^{5}$ cells/mL the day before transfection. Then, cells in 15 mL of medium were transferred to a T75 cell culture flask and incubated overnight at 37 ${}^{\circ }$ C in an incubator with 5% CO ${}_{2}$. When the cells reached 70–90% of confluence, the medium was discarded and 15 mL of G*ΔG-VSV virus (VSV-G pseudotyped virus, Kerafast) at a concentration of $7\times 10^{4}$ TCID ${}_{50}$/mL was used for infection. At the same time, the cells were transfected with 30 μg of S protein expression plasmid according to the user manual and then cultured in an incubator with 5% CO ${}_{2}$ at 37 ${}^{\circ }$ C. The cell supernatant was discarded after 6–8 h and 15 mL of fresh complete DMEM was added to the T75 cell culture flask, and after 24 h of culture in an incubator at 37 ${}^{\circ }$ C and 5% CO ${}_{2}$, the supernatant containing pseudotyped virus-containing culture supernatant was harvested, filtered, and frozen at $-80^{\circ }$ C for further use.

The reduction of luciferase gene expression in the pseudotyped virus infection assay was detected to evaluate the infection inhibition effect of ACE2 mutant decoy receptors and monoclonal antibodies. The tested samples were diluted (starting with 300 nM for C4-1 and monoclonal antibodies, 30-fold dilution for sera and then three-time gradient, a total of eight gradients), after which 50 μL of virus solution was added. On each 96-well plate, eight virus control wells (no test sample but only virus solution was added) and eight cell control wells (only a complete culture medium was added) were arranged. The 96-well plates were incubated at 37 ${}^{\circ }$ C for 1 h, after which trypsinized ACE2 expressing HEK-293T cells ( $2\times 10^{4}$/100 μL) were added to each well. After incubation for 24 h in an atmosphere comprising 5% CO ${}_{2}$ at 37 ${}^{\circ }$ C, chemiluminescence detection was conducted. To each well, 100 μL of luciferase substrate (PerkinElmer) was added, incubated at room temperature for 2 min, and then transferred to the detection whiteboard and measured using a luminometer (PerkinElmer). Each group contained two replicates. The EC ${}_{50}$ value of each sample was calculated using the Reed–Muench method.

## Supplementary Material

Supplemental Material
