# Supplementary material for: ACE2 decoy receptor generated by high-throughput saturation mutagenesis efficiently neutralizes SARS-CoV-2 and its prevalent variants
Source: Emerg Microbes Infect. 2022 Jun 1;11(1):1488–99. doi: 10.1080/22221751.2022.2079426 (PMC9176695; doi:10.1080/22221751.2022.2079426)
Supplement: Supplemental Material [file TEMI_A_2079426_SM3723.zip › Supplementary Materials/Supplementary Materials.docx]

Supplemental Materials for

## ACE2 Decoy Receptor Obtained by a High-throughput Saturation Mutagenesis Approach Efficiently Neutralizes SARS-CoV-2 and its prevalent variants

Bolun Wang^a*^, Junxuan Zhao^a*^, Shuo Liu^b*^, Jingyuan Feng^c^, Yufeng Luo^a^, Xinyu He^a^, Yanmin Wang^a^, Feixiang Ge^a^, Junyi Wang^a^, Buqing Ye^a^, Weijin Huang^a^, Xiaochen Bo^d^, Youchun Wang^b^ and Jianzhong Jeff Xi^a^

^a^Department of Biomedical Engineering, Peking University, Beijing, 100871, China

^b^Division of HIV/AIDS and Sex-transmitted Virus Vaccines, Institute for Biological Product Control, National Institutes for Food and Drug Control (NIFDC), Beijing 102629, China

^c^College of Chemistry, University of California Berkeley, Berkeley, California, United States

^d^Institute of Health Service and Transfusion Medicine, Beijing, 100850, China

Correspondence: Prof. Jianzhong Jeff Xi: [jzxi@pku.edu.cn](mailto:jzxi@pku.edu.cn) and Prof. Youchun Wang: wangyc@nifdc.org.cn

**Supplementary Figures**

**Supplementary Figure 1.** A novel method for high-throughput oligo synthesis of full-length mutation primers.

**Supplementary Figure 2.** (A) Ratios of amino acid mutations on ACE2-PD per plasmid based on a Sanger sequencing result of 22 pWSLV03 ACE2 mutation library plasmids; (B) Cell line selection based on the ratio of cell line endogenous expression of ACE2 to stably transfected total expression of ACE2. The ratio was determined by qRT-PCR. Data are mean ± SEM, n = 2 replicates.

**Supplementary Figure 3.** Fluorescent microscope image of ACE2-expressing HEK-293T cells in efficient binding with S-RBD-mFC-FITC, taken after 30 minutes of protein-cell incubation at 37 °C, scale bar = 100 μm.

**Supplementary Figure 4.** Examples showcasing the method for calculating enrichment scores.

**Supplementary Figure 5.** Comparison of median fitness scores for positions in alpha helices, beta strands, or unstructured regions. Alpha helix and beta strand assignments obtained through STRIDE for structure PDB: 6M0J. Unpaired t test was used to analyze differences between groups. *p < 0.05,**p < 0.01.

**Supplementary Figure 6.** Fitness scores for residues with important phenotypes on binding affinity. (A) Interacting residues and (B) Glycan sites.

**Supplementary Figure 7.** Enrichment scores for glycan-introducing mutations. Mutations on interacting sites were colored pink. Mutations at V212 and Y510 have different enrichment effects when mutated to T and S, indicating that enrichment at these positions are unlikely caused by glycosylation.

**Supplementary Figure 8.** The positions of selected mutant positions on the proteins.

**Supplementary Figure 9.** Binding of single amino acid substitution ACE2s expressed on HEK-293T for SARS- CoV-2 RBD. Data were normalized by WT-ACE2 RBD binding fluorescence. Data are mean ± SEM, n = 2 replicates. Unpaired t test was used to analyze differences between groups. *p < 0.05.

**Supplementary Figure 10.** Immobilized RBD association (t = 0 to 180 s) and dissociation (t > 180 s) with C3-1 (A), C3-2 (B), C3-3 (C), C5 (D), C4-1 (E), C4-2 (F) and WT-sACE2-Fc (G) measured by surface plasma resonance.

**Supplementary Figure 11.** The entry of SARS-CoV-2 and its variants into cell lines expressing hACE2 was blocked with soluble ACE2s (left, WT-sACE2; right, C4-1).

#####

**Supplementary Figure 12.** The entry of SARS-CoV-2 and its variants into cell lines expressing hACE2 was blocked with monoclonal antibodies. All results were shown as mean ± SEM. In each experiment, the infection assay was performed in duplicate wells.

**Supplementary Figure 13.** The entry of SARS-CoV-2 variants into cell lines expressing hACE2 was blocked by combinations of C4-1 and REGEN monoclonal antibodies (A) and C4-1 and bamlanivimab/etesevimab monoclonal antibodies (B). All results were shown as mean ± SEM. In each experiment, the infection assay was performed in duplicate wells.

##

**Supplementary Figure 14.** The entry of SARS-CoV-2 variants into cell lines expressing hACE2 was blocked by serum and serum/C4-1 mixture against SARS-CoV-2 D614G spike psedotyped virus (A) and omicron spike psedotyped virus (B). All results were shown as mean ± SEM. In each experiment, the infection assay was performed in duplicate wells.

## Supplementary Tables

Supplementary Table 1. Kinetic screen for WT-sACE2-Fc and sACE2-Fc variants in this study.

| Variant | ka (1/Ms) | kd (1/s) | KD (M) |
| --- | --- | --- | --- |
| C3-1 | 7.41 × 10^4^ | 6.88 × 10^-5^ | 9.28 × 10^-10^ |
| C3-2 | 9.96 × 10^4^ | 5.31 × 10^-5^ | 5.33 × 10^-10^ |
| C3-3 | 1.37 × 10^5^ | 7.87 × 10^-5^ | 5.75 × 10^-10^ |
| C4-1 | 1.05 × 10^5^ | 4.68 × 10^-5^ | 4.43 × 10^-10^ |
| C4-2 | 1.01 × 10^5^ | 4.62 × 10^-5^ | 4.59 × 10^-10^ |
| C5 | 1.25 × 10^5^ | 7.47 × 10^-5^ | 5.97 × 10^-10^ |
| WT sACE2-Fc | 8.25 × 10^4^ | 2.59 × 10^-4^ | 3.13 × 10^-9^ |

ka (binding rate constant), kd (disassociating rate constant) and KD (equilibrium constant for dissociation reaction) were calculated based on gradient dilutions of each protein.

Supplementary Table 2. ACE2 decoy receptors from this work and others’ works that showed enhanced SARS-CoV-2 spike binding affinity.

| **ACE2 decoy receptor** | **A25** | **T27** | **D30** | **K31** | **N33** | **H34** | **E35** | **Q42** | **L79** | **N90** | **L91** | **T92** | **N322** | **Q325** | **N330** | **H345** | **A386** | **K475** | **Additional mutations** | **Reference** |
| --- | --- | --- | --- | --- | --- | --- | --- | --- | --- | --- | --- | --- | --- | --- | --- | --- | --- | --- | --- | --- |
| C3-1 |  |  | D30I |  |  |  |  |  | L79W |  |  |  |  |  |  |  |  | K475F |  | This work |
| C3-2 |  |  | D30I |  |  |  |  |  | L79W |  |  | T92N |  |  |  |  |  |  |  |  |
| C3-3 |  |  | D30I |  |  |  |  |  | L79W |  |  |  | N322V |  |  |  |  |  |  |  |
| C4-1 |  |  | D30I |  |  |  |  |  | L79W |  |  | T92N |  |  |  |  |  | K475F |  |  |
| C4-2 |  |  | D30I |  |  |  |  |  | L79W |  |  |  | N322V |  |  |  |  | K475F |  |  |
| C5 |  |  | D30I |  |  |  |  |  | L79W |  |  | T92N | N322V |  |  |  |  | K475F |  |  |
| Chan-v1 |  |  |  |  |  | H34A |  |  |  |  |  | T92Q |  | Q352P |  |  | A386L |  |  | Reference 16 |
| Chan-v2 |  | T27Y |  |  |  |  |  |  | L79T |  |  |  |  |  | N330Y |  | A386L |  |  |  |
| Chan-v2.1 |  |  |  |  |  |  |  |  | L79T |  |  |  |  |  | N330Y |  | A386L |  |  |  |
| Chan-v2.2 |  | T27Y |  |  |  |  |  |  |  |  |  |  |  |  | N330Y |  | A386L |  |  |  |
| Chan-v2.3 |  | T27Y |  |  |  |  |  |  | L79T |  |  |  |  |  |  |  | A386L |  |  |  |
| Chan-v2.4 |  | T27Y |  |  |  |  |  |  | L79T |  |  |  |  |  | N330Y |  |  |  |  |  |
| Chan-v3 | A25V | T27Y |  |  |  |  |  |  |  |  |  | T92Q |  | Q325P |  |  | A386L |  |  |  |
| Chan-v4 |  |  |  |  |  | H34A |  |  | L79T |  |  |  |  |  | N330Y |  | A386L |  |  |  |
| Chan-v5 | A25V |  |  |  |  |  |  |  |  |  |  | T92Q |  |  |  |  | A386L |  |  |  |
| Chan-v6 |  | T27Y |  |  |  |  |  | Q42L | L79T |  |  | T92Q |  | Q325P | N330Y |  | A386L |  |  |  |
| Glasgow-14 |  |  |  |  |  | H34V |  |  |  |  |  |  |  |  |  |  |  |  |  | Reference 17 |
| Glasgow-19 |  |  |  | K31F |  | H34I | E35Q |  |  |  |  |  |  |  |  |  |  |  |  |  |
| Glasgow-118 |  |  |  |  |  | H34V |  |  |  | N90Q |  |  |  |  |  |  |  |  | H374N, H378N |  |
| Glasgow-310 | A25V | T27Y |  |  |  | H34A |  |  |  |  |  |  |  |  |  | H345L |  |  | F40D |  |
| Glasgow-311 |  |  |  | K31Y |  |  |  |  | L79T |  | L91P |  |  |  |  | H345L |  |  | W69V |  |
| Glasgow-313 |  |  |  | K31F | N33D | H34S | E35Q |  |  |  |  |  |  |  |  | H345L |  |  |  |  |
| Glasgow-353 |  | T27A |  | K31F | N33D | H34S | E35Q |  | L79P |  |  |  |  |  |  | H345L |  |  | N61D, K68R |  |
| Higuchi-3N39v2 | A25V |  |  | K31N |  |  | E35K |  | L79F |  |  |  |  |  |  |  |  |  |  | Reference 18 |
| Higuchi-3J113v2 |  |  |  | K31M |  |  | E35K |  | L79F |  |  |  |  |  |  |  |  |  | Q60R |  |
| Higuchi-3J320v2 |  |  |  |  |  | H34A |  |  |  |  |  | T92Q |  |  |  |  |  |  | T20I, Q101H |  |
| Sims-CDY01 |  |  |  | K31M |  |  | E35K | Q42R |  |  | L91P |  |  |  | N330Y |  |  |  |  | Reference 45 |
| Sims-CDY03 |  |  |  | K31R |  |  | E35V |  | L79F | N90D |  |  |  |  | N330Y |  |  |  | L39P, V59A |  |
| Sims-CDY05 |  |  |  | K31M |  |  | E35K |  | L79I |  | L91P |  |  |  | N330Y |  |  |  |  |  |
| Sims-CDY09 |  |  |  | K31M |  |  | E35K |  | L79F |  | L91P |  |  |  | N330Y |  |  |  |  |  |
| Sims-CDY14 |  |  |  | K31M |  |  | E35K |  | L79F |  | L91P |  |  |  | N330Y |  |  |  | S47A |  |

20
